# Study on the Structure of a Mixed KCl and K_2_SO_4_ Aqueous Solution Using a Modified X-ray Scattering Device, Raman Spectroscopy, and Molecular Dynamics Simulation

**DOI:** 10.3390/molecules27175575

**Published:** 2022-08-30

**Authors:** Mengdan Qiao, Fei Li, Xianze Meng, Meiling Wang, Hanyu Zhu, Zhiyong Ji, Yingying Zhao, Jie Liu, Shizhao Wang, Xiaofu Guo, Jingtao Bi, Junsheng Yuan

**Affiliations:** 1 Engineering Research Center of Seawater Utilization of Ministry of Education, School of Chemical Engineering and Technology, Hebei University of Technology, Tianjin 300130, China; 2 School of Materials, Sun Yat-sen University, Guangzhou 511400, China

**Keywords:** KCl, K_2_SO_4_, X-ray diffraction, Raman spectroscopy, molecular dynamics simulations, solution structure

## Abstract

The microstructure of a mixed KCl and K_2_SO_4_ aqueous solution was studied using X-ray scattering (XRS), Raman spectroscopy, and molecular dynamics simulation (MD). Reduced structure functions [*F*(*Q*)], reduced pair distribution functions [*G*(*r*)], Raman spectrum, and pair distribution functions (PDF) were obtained. The XRS results show that the main peak (r = 2.81 Å) of *G*(*r*) shifted to the right of the axis (r = 3.15 Å) with increased KCl and decreased K_2_SO_4_. The main peak was at r = 3.15 Å when the KCl concentration was 26.00% and the K_2_SO_4_ concentration was 0.00%. It is speculated that this phenomenon was caused by the main interaction changing, from K-O_W_ (r = 2.80 Å) and O_W_-O_W_ (r = 2.80 Å), to Cl^−^-O_W_ (r = 3.14 Å) and K^+^-Cl^−^ (r = 3.15 Å). According to the trend of the hydrogen bond structure in the Raman spectrum, when the concentration of KCl was high and K_2_SO_4_ was low, the destruction of the tetrahedral hydrogen bond network in the solution was more serious. This shows that the destruction strength of the anion to the hydrogen bond network structure in solution was Cl^−^ > SO_4_^2−^. In the MD simulations, the coordination number of O_W_-O_W_ decreased with increasing KCl concentration, indicating that the tetrahedral hydrogen bond network was severely disrupted, which confirmed the results of the Raman spectroscopy. The hydration radius and coordination number of SO_4_^2−^ in the mixed solution were larger than Cl^−^, thus revealing the reason why the solubility of KCl in water was greater than that of K_2_SO_4_ at room temperature.

## 1. Introduction

Potassium is essential for production and life [1,2,3,4]; with the rapid development of the economy in China and the need for agricultural production, the demand for potassium has become increasingly strong. However, potassium resources are relatively scarce in China, and global terrestrial potash resources are limited. An efficient potassium extraction process is important. It is hoped that this solution structure study can give some theoretical advice on the development of this process.

As far as potash solution is concerned, some scholars studied it as early as the middle of the last century. Gallo and Corradini [5] used molecular dynamics to study the structural properties of aqueous potassium chloride and fluoride solutions under ambient and supercooled conditions. It was found that the addition of both KCl and KF resulted in the distortion of the O-O structure and the second shell would move to a shorter distance. This effect was more pronounced as the concentration increased and more pronounced in KF than in KCl, especially with supercooling. Kerdcharoen [6] used QM/MM and ONIOM-XS methods to study K^+^ and Ca^2+^ in water and calculated that the average hydration number around K^+^ was 6.3. Kabbalee [7] also used ONIOM-XS MD simulation to study the solvation structure and kinetics of K^+^ in ammonia water. Simulations showed that the first solvation shell of K^+^ is very flexible and can form 4- to 10-fold coordinated K^+^ ligands. The average coordination number of K^+^ is 7.0, and it contains about 4.8 water molecules and 2.2 ammonia molecules. Our group has also conducted some studies on the structure of potassium salt solutions [8,9,10,11]. For example, by studying a mixed solution of KCl and NaCl [12], it was found that Na^+^ damages the hydrogen bond structure to a greater degree than K^+^. Under the experimental conditions, both the radius of hydration and the number of hydrations of Na^+^ were smaller than that of K^+^, which provided a micro reason to understand the solubility difference between these two salts. For KCl aqueous solution, as the concentration of the solution increases, contact ion pairs appear when the concentration is higher than 15.00%, and the hydration number of K^+^ gradually decreases [13]. Experimental and simulation [8,9,10, 11,12,13,14,15,16] analyses showed that the coordination number of K^+^ is in the range of 5.71–6.53 at the temperature of 300–450 K and the concentration of 0.01–3 mol/L.

Studying the structure of a KCl and K_2_SO_4_ mixed aqueous solution has an important practical significance for guiding the industrial extraction of potassium from seawater. Generally speaking, an X-ray scattering experiment is one of the most effective methods for studying the structure of an aqueous solution. However, due to the complexity of the structure of the aqueous solution, results obtained using only one test method are not convincing. Therefore, a combination of multiple test methods is required, to verify the accuracy of the results. Many scholars have studied the OH stretching vibration of water using the Raman spectrum [17,18,19,20,21] and proposed that the Raman OH stretching band can be fitted into five sub-bands and that each peak can be assigned to OH groups with different local hydrogen bond structures. Therefore, the microstructure changes of mixed aqueous solutions of KCl and K_2_SO_4_ can be inferred from the interpretation of the OH stretching zone of water.

In this work, the effects of KCl and K_2_SO_4_ on a mixed aqueous solution structure were studied using a self-modified X-ray scattering device, Raman spectroscopy, and molecular dynamics simulation. In this study, we tried to use several test methods, to obtain a more comprehensive solution of the microstructure information, which can be used as a basis for judging the changes of ionic interactions in a solution and to provide theoretical guidance for their separation.

## 2. Results and Discussion

### 2.1. X-ray Scattering Analysis

**Figure 1:**
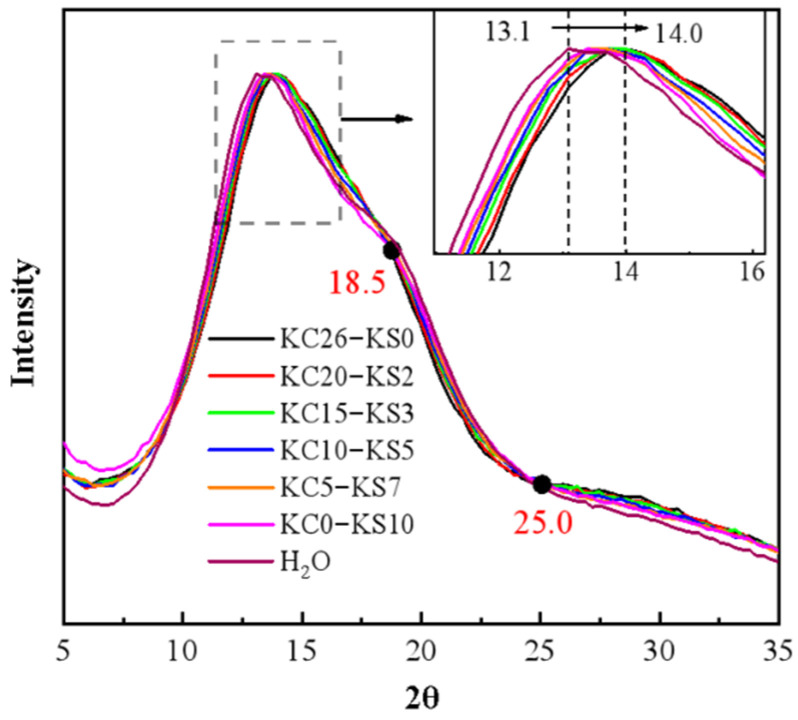
X-ray scattering spectra of mixed KCl and K_2_SO_4_ aqueous solutions with different mass fractions.

**Figure 2:**
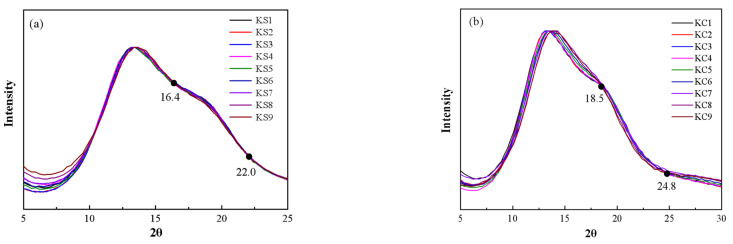
Local magnification of X-ray scattering in a single aqueous solution system: (**a**) K_2_SO_4_ aqueous solution; (**b**) KCl aqueous solution.

The normalized results of the X-ray scattering of the mixed aqueous solution of KCl and K_2_SO_4_ are shown in Figure 1. It can be seen that as the mass fraction of K_2_SO_4_ in the solution decreased and the mass fraction of the KCl increased, the characteristic peak gradually shifted from 13.1° to 14.0°; and the shoulder peak at 19° gradually disappeared. Comparing the scattering spectrum of the mixed solution and pure water, it can be seen that the scattering spectrum of pure water was closer to that of the 10.0% K_2_SO_4_ solution, but it was quite different from the scattering spectrum of 26.0% KCl solution. It is speculated that the reason for this is that the quantity ratio of ions to water molecules in the 26.0% KCl solution (1:11.8) was much greater than the quantity ratio in the 10.0% K_2_SO_4_ solution (1:87). In addition, the curve intersects at 18.5° and 25.0°. Through the study of a single aqueous solution, it can be seen from Figure 2 that, in the K_2_SO_4_ solution, the spectrum has intersection points at approximately 16.4° and 22.0°; while in the KCl solution, the intersection points appear at 18.5° and 24.8°. It can be seen that KCl had a great influence on the mixed solution system. This is because the solubility of K_2_SO_4_ (12 g/100 g H_2_O at 25 °C) was lower than that of KCl (35.7 g/100 g H_2_O at 25 °C).

**Figure 3:**
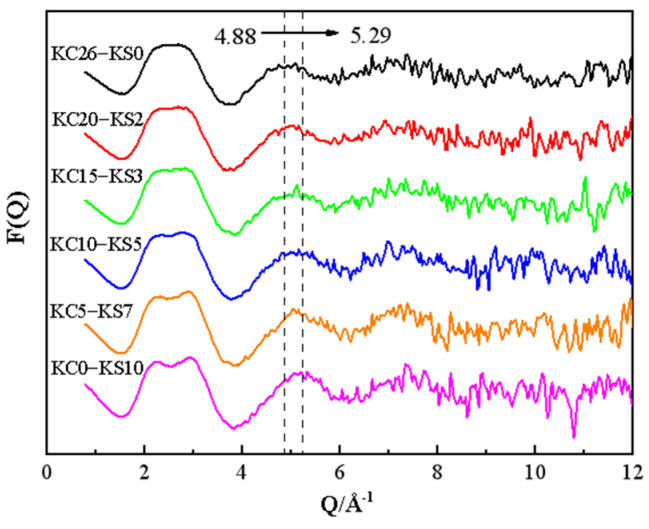
*F*(*Q*) of mixed KCl and K_2_SO_4_ aqueous solution with different mass fractions.

**Figure 4:**
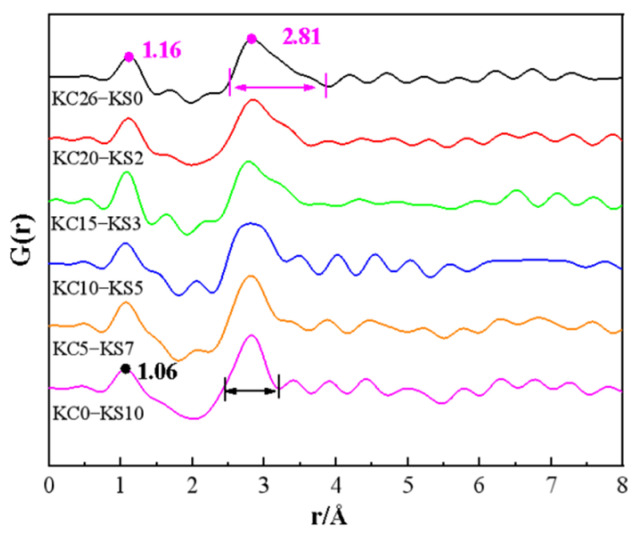
*G*(*r*) of mixed KCl and K_2_SO_4_ aqueous solution with different mass fractions.

The reduced structure function [*F*(*Q*)] and the reduced pair distribution functions [*G*(*r*)] of the mixed aqueous solution system obtained using PDFgetX3 processing are shown in Figure 3 and Figure 4.

It can be seen from Figure 3 that with the decrease of KCl mass fraction and the increase of K_2_SO_4_ mass fraction in the mixed solution, a flat-topped peak appeared near Q = 2.5 Å^−1^, which gradually split into two peaks, and the double peaks became more and more obvious. The peak appearing at this position is the structural characteristic peak of the aqueous solution, which is related to the hydrogen bond network structure in the water [22]. The peak near Q = 5 Å^−1^, which is very sensitive to the degree of hydrogen bond destruction in the solution [23], tended to move to the right of abscissa. Study of the structure of a single potassium salt aqueous solution shows that the law of peak movement in the mixed aqueous solution is the result of the superposition of two potassium salts, but the effect of KCl is stronger.

At the same time, an *G*(*r*) image of mixed the KCl and K_2_SO_4_ aqueous solutions is presented in Figure 4. It can be seen from Figure 4 that, as the mass fraction of KCl increased and the mass fraction of K_2_SO_4_ decreased, the peak near r = 2.81 Å gradually broadened. It can be seen from a single aqueous solution that the combined action of O-O and K^+^-O is related to this peak. In the KCl aqueous solution with a high mass fraction, the main peak appeared at 3.15 Å [13]. It is judged that, in the mixed solution, K^+^-Cl^−^ contact ion pairs appeared with the increase of KCl mass fraction, resulting in the broadening of the peak shape near r = 2.81 Å. The peak near 1.0 Å represents the intramolecular O-H interaction of water molecules. As can be seen from Figure 4, as the mass fraction of KCl in the mixed solution increased, the peak value moved to the right on the abscissa, from 1.06 Å to 1.16 Å, which is consistent with the change rule of a single KCl aqueous solution. This proves that the influence of KCl on the structure of the aqueous solution was greater than K_2_SO_4_. At the same time, it also shows that the influence of Cl^−^ on the structure of the aqueous solution was stronger than that of SO_4_^2−^. For the mixed aqueous solution system, this peak position was greater than 0.97 Å calculated by molecular dynamics, which is the O–H covalent bond length in the pure water molecule [13]. This shows that the addition of KCl and K_2_SO_4_ both made the O-H covalent bond lengthen and have a stretching tendency. To discuss the generation of contact ion pairs and the changes in hydrogen bonds in more detail, it is worthwhile to analyze the results of the Raman spectroscopy and molecular dynamics simulations.

### 2.2. Raman Spectroscopy Results and Analysis

**Figure 5:**
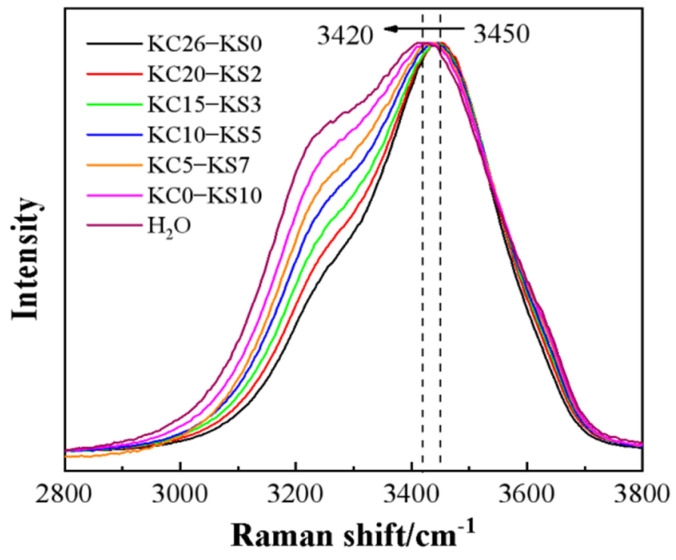
Raman spectra of mixed KCl and K_2_SO_4_ aqueous solution with different mass fractions.

In order to make the comparison more convenient, the Raman intensity has been normalized, and the results are shown in Figure 5.

It can be seen from Figure 5 that, with the decrease of KCl and the increase of K_2_SO_4_ in the mixed aqueous solution, the shoulder peaks appearing in the range of 3200–3300 cm^−1^ showed an expanding trend; the overall peak shape of the scanning range gradually broadened. At the same time, the characteristic peak shifted significantly, from 3450 cm^−1^ to 3424 cm^−1^. Through the study of the structure of a single potassium salt solution, it can be proven that these changes are consistent with the laws presented in a single potassium salt aqueous solution [24].

**Figure 6:**
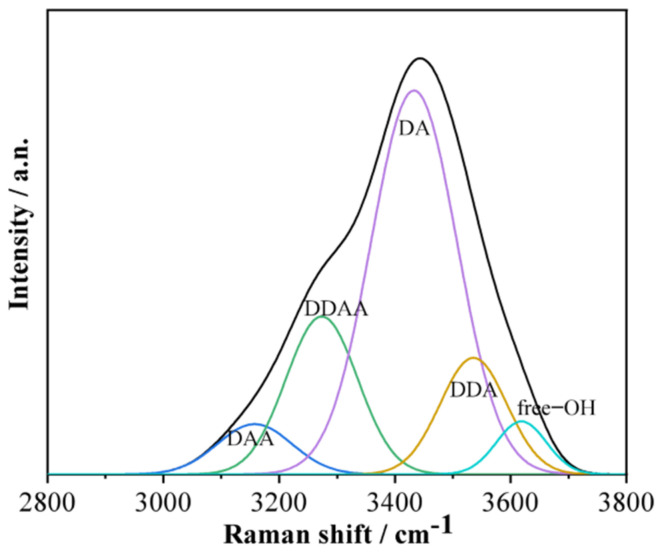
Deconvolution Fitting of Raman Spectra of 5% KCl-7.1% K_2_SO_4_ Mixed Solution.

**Figure 7:**
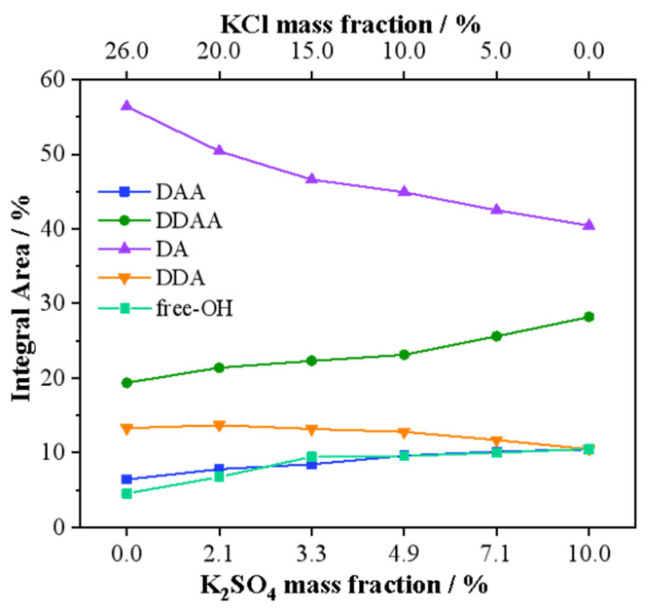
Variation of the Raman peak area in the OH stretching vibration range of KCl–K_2_SO_4_ mixed aqueous solutions with different mass fractions.

To further explore the microstructure of the mixed aqueous solution of KCl and K_2_SO_4_, it was divided into five Gaussian peaks using a deconvolution fitting method [25,26,27], which were assigned to υ_DAA-OH_, υ_DDAA-OH_, υ_DA-OH_, υ_DDA-OH_, and υ_free-OH_ symmetrical stretching vibrations, and the results are shown in Figure 6. Where D stands for the donor, which provides H to form hydrogen bonds with other water molecules; and A is the acceptor, which bonds with other water molecules using lone pair electrons on oxygen. Figure 7 shows the proportion of the five hydrogen bond structures in the mixed aqueous solution as a function of the mass fraction. These changes indicated that the content of DDAA and free OH increased continuously as the Cl^−^ concentration gradually decreased and the SO_4_^2−^ concentration gradually increased in the mixed aqueous solution; in this process, the DDA and DA-type hydrogen bonds continued to decrease. From the changing trend of the DDAA-type hydrogen bond structure in Figure 7, it can be seen that the destruction of the DDAA-type hydrogen bond structure by Cl^−^ was greater than that of SO_4_^2−^. Therefore, the changing trend of the mixed aqueous solution was mainly affected by the change in the KCl mass fraction.

Table 1 lists the Gaussian peak positions of different types of OH stretching vibrations in mixed solutions. Table 1 demonstrates that the peak position of each Gaussian peak was reasonably stable and did not fluctuate significantly over a wide range of wavenumbers as the solute concentration was altered.

### 2.3. Simulation Results Analysis

Pair distribution function (PDF) is a method to obtain the atomic structure characteristics of a system in molecular dynamics simulation. We used PDF to further analyze the microstructure of the mixed aqueous solution. The hydration radius can be obtained directly from the PDF, where the first peak corresponds to the first coordination layer around the central atom. The first peak in PDF corresponds to the first coordination layer surrounding the central atom. The number of atoms in the first coordination layer (coordination number) is the product of the integral of the area of the first peak and the number density. According to Equation (1):

(1) $$N(r)=4\pi \rho_{N}\int_{0}^{r}r^{2}g(r)dr,$$

where *ρ_N_* represents the average number density, and *r* represents the hydration radius.

**Figure 8:**
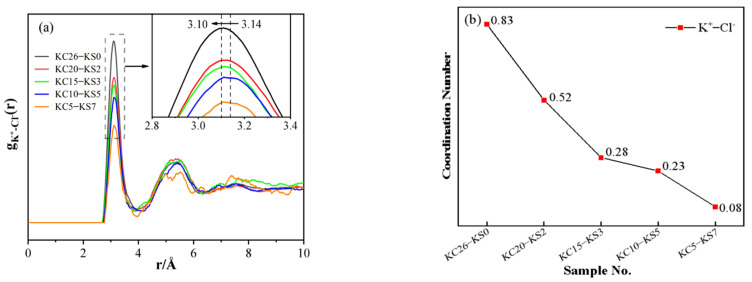
PDF (**a**) and coordination number (**b**) of K^+^-Cl^−^ in different concentrations of KCl–K_2_SO_4_ aqueous solution.

Figure 8 depicts the PDF and coordination number diagrams for K^+^-Cl^−^ in mixed aqueous solutions containing varying concentrations of KCl and K_2_SO_4_. It can be seen from Figure 8a that the first peak was relatively strong, indicating the short-range order of the ion arrangement in the aqueous solution. As can be seen from the changes in PDF and the coordination number in Figure 8, as the mass fraction of KCl in the mixed aqueous solution increased, the first peak in (a) moved to a smaller distance from 3.14 Å to 3.10 Å; (b) the coordination number between K^+^-Cl^−^ increased, from 0.08 to 0.83, indicating an enhanced interaction between K^+^-Cl^−^. This conclusion confirms that in the *G*(*r*) function, the broadening of the peak at the 2.81 Å position toward the high r direction was caused by the K^+^-Cl^−^ interaction.

**Figure 9:**
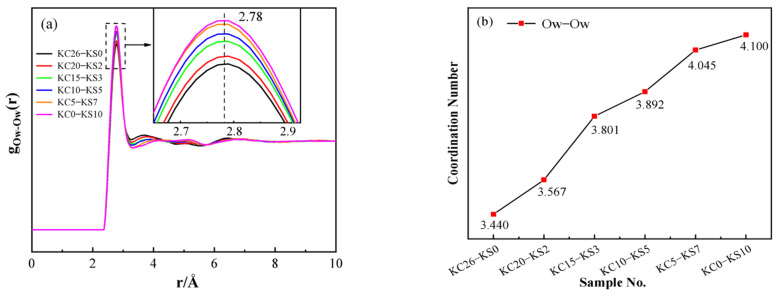
PDF (**a**) and coordination number (**b**) of O_W_-O_W_ in different concentrations of KCl–K_2_SO_4_ aqueous solution.

Figure 9 depicts the PDF and coordination number plots of O_W_-O_W_ in mixed aqueous solutions of KCl and K_2_SO_4_ with different concentrations. It can be seen from Figure 9 that the radial distribution function and coordination number of O_W_-O_W_ in different concentrations of KCl-K_2_SO_4_ aqueous solutions showed regular changes. It can be seen from Figure 9b that, the higher the KCl concentration, the lower the coordination number, ranging from 4.10 to 3.44, indicating that the O_W_-O_W_ interaction in water was gradually weakened, and the tetrahedral hydrogen-bonded water network was severely damaged. This is consistent with the Raman conclusion that KCl damages the hydrogen bond structure of DDAA more severely than K_2_SO_4_.

**Figure 10:**
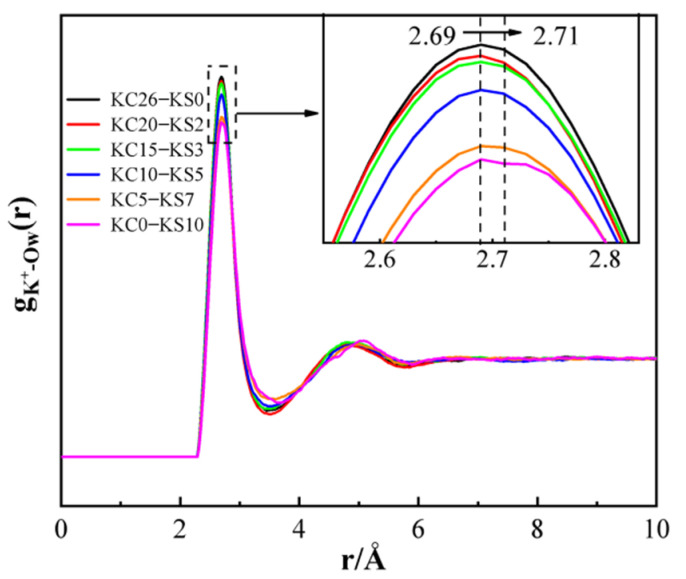
PDF of K^+^-O_W_ in different concentrations of KCl–K_2_SO_4_ aqueous solution.

Figure 10 is a PDF diagram of K-O_W_ with various concentrations of KCl and K_2_SO_4_ in mixed aqueous solutions. Figure 10 demonstrates that the first peak of the K^+^-O_W_ PDF was at 2.7 Å. Combining the positions of the first peaks in Figure 8a and Figure 9a, it was proven that the earlier *G*(*r*) analysis hypotheses, that the peak near 2.8 Å resulted from the interaction of K^+^-Cl^−^, K^+^-O_W_, and O_W_-O_W_, were accurate.

**Figure 11:**
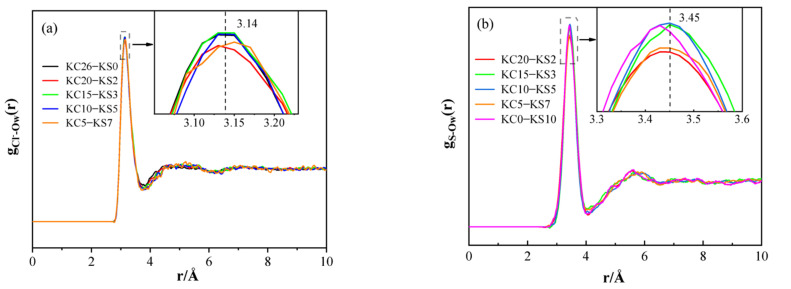
PDF of Cl^−^-O_W_ (**a**) and S-O_W_ (**b**) of KCl–K_2_SO_4_ aqueous solutions.

Figure 11 shows the PDF of Cl^−^-O_W_ and S-O_W_ in mixed aqueous solutions of KCl and K_2_SO_4_ with different concentrations. It can be seen from Figure 11 that the first peak functions of the radial distributions of Cl^−^-O_W_ and S-O_W_ appeared around 3.14 Å and 3.45 Å, respectively, and the first peak of S-O_W_ was higher than that of Cl^−^-O_W_. In addition, the position of the first peak did not change much in the mixed aqueous solutions with different mass fractions, indicating that the concentration had little effect on the interaction between Cl^−^-O_W_ and S-O_W_.

**Figure 12:**
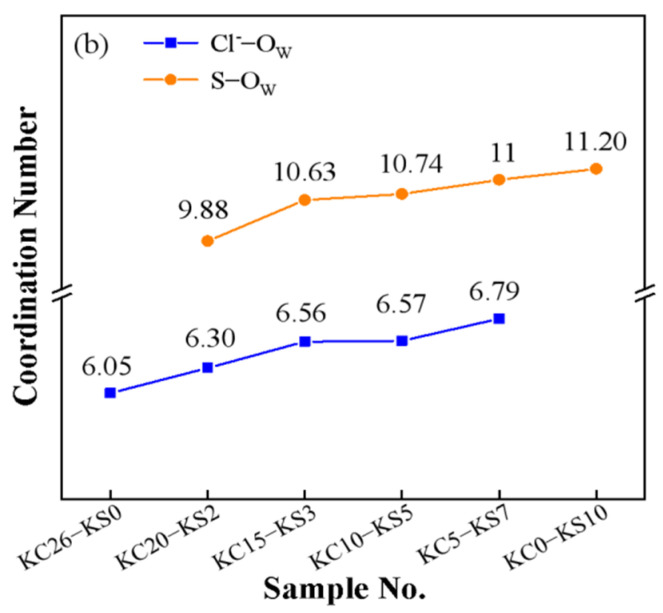
Coordination number of Cl^−^-O_W_ (blue) and S-O_W_ (red) in mixed aqueous solutions.

Figure 12 is a diagram of the coordination numbers of Cl^−^-O_W_ (blue) and S-O_W_ (red) in mixed aqueous solutions. It can be seen from the figure that the coordination number of Cl^−^-O_W_ was smaller than that of S-O_W_. Combining the PDF diagrams of Cl^−^-O_W_ and S-O_W_, it was found that the hydration radius and coordination number of SO_4_^2^^-^ in the mixed solution components was larger than that of Cl^−^, thus revealing the reason why the solubility of KCl in water was greater than that of K_2_SO_4_.

## 3. Materials and Methods

### 3.1. Sample Preparation

KCl and K_2_SO_4_ were used to prepare the solution. GR reagent produced by Tianjin Guangfu Fine Chemical Research Institute (the contents of KCl and K_2_SO_4_ are not less than 99.99%) was selected. The experimental water was prepared using the uhw-i90t, and its resistivity was 18.25 MΩ cm at room temperature. The mixed aqueous solutions of KCl and K_2_SO_4_ with mass fractions of 26.0% KCl/0.0% K_2_SO_4_ (abbreviated as “KC26-KS0”, the same below); 20.0% KCl/2.1% K_2_SO_4_; 15.0% KCl/3.3% K_2_SO_4_; 10.0% KCl/4.9% K_2_SO_4_; 5.0% KCl/7.1% K_2_SO_4_; and 0.0% KCl/10.0% K_2_SO_4_ were prepared by the mass method. The prepared mixed solutions with their basic properties are shown in Table 2.

To compare the effect of single solutions, the experiments were also configured with different concentrations of single K_2_SO_4_ aqueous solution and single KCl aqueous solution, which can be seen in Table 3 and Table 4.

### 3.2. X-ray Scattering Experiment

X-ray scattering data were tested on a modified D8–Focus X-ray scattering device [28]. The light energy was 17.45 keV, and the 2θ scanning range was 5°–150°.

### 3.3. Raman Spectroscopy Experiment

Raman spectroscopy was performed on a confocal Raman microscope spectrometer (inVia, Renishaw, London, UK) at room temperature. The laser excitation wavelength was 532 nm, the objective lens was 50 times, and the spectral scanning range was 2800–3800 cm^−1^.

### 3.4. Molecular Dynamics Simulation

The simulations in this paper used the potential energy model provided by the COMPASS II force field of the Materials Studio software package [29,30]. The Construction function in the Amorphous Cell module was used to build a preliminary model. Then, on this basis, the Geometry Optimization function in the Forcite Calculation module was used to perform ensemble optimization on the preliminary model; and the force field was the COMPASS II force field (See Supplementary Materials for details). The number of particles used was determined by the solution concentration, as shown in Table 2. The Nose method was used for the isothermal simulation, the simulation temperature was 298 K, the side length of the initial water box model was 26 Å, and the Berendsen method was selected for the pressure control. Three specification sets of NVT, NPT, and NVE were used in sequence. The initial configuration of the simulation was a face-centered cubic lattice, and the initial orientation of each particle was random. The initial velocity of each particle was sampled according to the Maxwell distribution, using cubic periodic boundary conditions. The van der Waals effect and electrostatic effect were obtained using the Atom-based method and the Ewald method, respectively; and the motion equation of the system was solved using the velocity-Verlet algorithm. The time step of the simulation was 0.2 fs, and the total time of each simulation was 100 ps.

### 3.5. Method of Structure Analysis

The intensity of the X-ray scattering spectrum was the superposition of multiple scattering intensities [31]. PDFgetX3 was used to process the X-ray scattering data, to obtain the information needed to analyze the structure of the solution [32]. The X-ray scattering results could be converted into the structural function (*S*(*Q*)) through this software [33]. Equation (2) was used to Fourier transform the *S*(*Q*), to obtain the reduced pair distribution function, *G*(*r*) [34].

(2) $$\begin{array}{cc} G(r) & =(2/\pi )\int_{0}^{\infty }Q\left[S(Q)-1\right]\sin(Qr)dQ\\ & =4\pi r\rho_{0}\left(g(r)-1\right)\\ & =(2/\pi )\int_{0}^{\infty }F(Q)\sin(Qr)dQ \end{array}$$

where F(Q)=Q[S(Q)−1], which is called the reduced structure functions. *Q* represents the scattering vector; *r* is the distance between atoms; *ρ*_0_ is the number density of atoms in the system; and *g*(*r*) is the atomic pair distribution function.

## 4. Conclusions

The microscopic solution structure of a KCl and K_2_SO_4_ mixed aqueous solution system was examined using X-ray scattering, Raman spectroscopy, and molecular dynamics simulation in this article, yielding the following results:

The X-ray scattering results showed that as KCl increased and K_2_SO_4_ decreased, the main peak of *G*(*r*) was widened to the right side of the abscissa. The main peak was wide, as the KCl concentration was 26.00% and the K_2_SO_4_ concentration was 0.00%. It is speculated that this phenomenon was due to the main interaction between K-O_W_ (r = 2.80 Å) and O_W_-O_W_ (r = 3.10 Å) and K^+^-Cl^−^ (r = 3.15 Å). The results of Raman spectroscopy demonstrated that the DDAA–type hydrogen bonding structure was disrupted, and the disruption of the DDAA–type hydrogen bonding structure by Cl^−^ was larger than that by SO_4_^2−^. In the MD simulation, the coordination number of O_W_-O_W_ decreased with the increase of KCl concentration, indicating that the tetrahedral hydrogen bond network was severely damaged, which confirmed the results of the Raman spectrum. In summary, it was shown that the trend of KCl-K_2_SO_4_ mixed aqueous solution was mainly influenced by the concentration of KCl.

## Figures and Tables

**Table 1:** The deconvolution parameters of Gaussian peaks in the OH stretching vibration region in KCl–K_2_SO_4_ mixed solutions with different mass fractions.

Sample No.	Raman Shift (cm^−1^)				
	DDA	DDAA	DA	DAA	Free OH
KC26-KS0	3157.6	3273.7	3433.0	3535.6	3618.5
KC20-KS2	3168.2	3271.7	3430.0	3525.6	3616.6
KC15-KS3	3157.8	3273.8	3427.4	3521.6	3607.2
KC10-KS5	3154.3	3269.5	3421.2	3512.0	3605.4
KC5-KS7	3151.7	3269.5	3422.5	3514.7	3606.2
KC0-KS10	3142.1	3266.0	3418.2	3514.7	3602.5

**Table 2:** Configurate information of KCl and K_2_SO_4_ mixed aqueous solution.

Sample No.	Mass Fraction/%		Density/g·cm^−3^	n_k_:n_c_:n_s_:n_h_ *
	KCl	K_2_SO_4_		
KC26-KS0	26.0	0.0	1.1698	52:52:0:609
KC20-KS2	20.0	2.1	1.1521	42:38:2:609
KC15-KS3	15.0	3.3	1.1267	29: 23:3:609
KC10-KS5	10.0	4.9	1.1054	25:17:4 609
KC5-KS7	5.0	7.1	1.0900	18:8:5:609
KC0-KS10	0.0	10.0	1.0641	14:0:7:609

* The number of ions and molecules in the box during the molecular dynamics simulation, using Construction modules of Materials Studio software. n_k_: K^+^; n_c_: Cl^−^; n_s_: SO_4_^2-^; n_h_: H_2_O.

**Table 3:** Configuration information of K_2_SO_4_ aqueous solutions.

Sample No.	K_2_SO_4_ Mass Fraction/%	Density/g·cm^−3^	n(K_2_SO_4_):n(H_2_O)
KS1	0.08	0.9928	1:12078.3
KS2	0.16	0.9931	1:10063.6
KS3	0.24	0.9943	1:8049.0
KS4	0.48	0.9964	1:2005.0
KS5	1.00	0.9989	1:957.4
KS6	2.50	1.0111	1:625.8
KS7	5.00	1.0315	1:183.7
KS8	7.50	1.0478	1:135.4
KS9	10.0	1.0641	1:87.0

**Table 4:** Configuration information of KCl aqueous solutions.

Sample No.	KCl Mass Fraction/%	Density/g·cm^−3^	n(KCl):n(H_2_O)
KC1	0.07	0.9966	1:5874.9
KC2	0.21	0.9969	1:1971.5
KC3	0.42	0.9985	1:983.4
KC4	1.00	1.0001	1:410.0
KC5	5.00	1.0258	1:78.5
KC6	10.00	1.0587	1:37.2
KC7	15.00	1.0923	1:23.5
KC8	20.00	1.1265	1:16.5
KC9	26.00	1.1698	1:11.8

## Data Availability

The datasets used and/or analyzed in the present study are available from the corresponding author upon reasonable request.

## Supplementary Materials

The supporting information [35,36,37,38,39,40,41] can be downloaded at: https://www.mdpi.com/article/10.3390/molecules27175575/s1.

## Sample Availability

Sample information for compounds is available from the authors.
