# Supplementary material for: Study on the Structure of a Mixed KCl and K2SO4 Aqueous Solution Using a Modified X-ray Scattering Device, Raman Spectroscopy, and Molecular Dynamics Simulation
Source: Molecules. 2022 Aug 30;27(17):5575. doi: 10.3390/molecules27175575 (PMC9457528; doi:10.3390/molecules27175575)
Supplement: Supplementary file 1 [file molecules-27-05575-s001.zip › molecules-1877167-supplementary.pdf]

Supporting information

Study on the structure of mixed KCl and K_2SO_4 aqueous solution by modified X-ray scattering device, Raman spectroscopy and molecular dynamics simulation

Molecular dynamics simulation

The COMPASS II force field is an extension to the COMPASS force field [1]. The functional form used in this force field is the same as that used in the COMPASS force field [2,3]:

$$E_{total} = \sum_b \left[k_2(b-b_0)^2 + k_3(b-b_0)^3 + k_4(b-b_0)^4\right] + \sum_\theta \left[k_2(\theta-\theta_0)^2 + k_3(\theta-\theta_0)^3 + k_4(\theta-\theta_0)^4\right] +$$
$$\sum_\phi \left[k_1(1-\cos\phi) + k_2(1-\cos 2\phi) + k_3(1-\cos 3\phi)\right] + \sum_\chi k_2\chi^2 + \sum_{b,b'} k(b-b_0)(b'-b'_0) +$$
$$\sum_{b,\theta} k(b-b_0)(\theta-\theta_0) + \sum_{b,\phi} (b-b_0)\left[k_1\cos\phi + k_2\cos 2\phi + k_3\cos 3\phi\right] + \sum_{\theta,\phi} (\theta-\theta_0)[k_1\cos\phi +$$
$$k_2\cos 2\phi + k_3\cos 3\phi] + \sum_{b,\theta} k(\theta'-\theta'_0)(\theta-\theta_0) + \sum_{\theta,\theta',\phi} k(\theta-\theta_0)(\theta'-\theta'_0)\cos\phi +$$
$$\sum_{i,j} \frac{q_i q_j}{r_{ij}} + \sum_{i,j} \varepsilon_{ij}\left[2\left(\frac{r_{ij}^0}{r_{ij}}\right)^9 - 3\left(\frac{r_{ij}^0}{r_{ij}}\right)^6\right]$$

The non-bond action potential form in the formula is：

$$E_{vdw} = \sum_{i,j} \varepsilon_{ij} \left[2\left(\frac{r_{ij}^{\ 0}}{r_{ij}} \right)^9 - 3\left(\frac{r_{ij}^{\ 0}}{r_{ij}} \right)^6 \right]$$

$$E_{coul} = \sum \frac{q_i q_j}{r_{ij}}$$

The LJ-9-6 parameters are given for like atom pairs. For unlike atom pairs, a 6th order combination law is used to calculate the off-diagonal parameters:

$$r_{i,j}^{0} = \left(\frac{\left(r_i^{0}\right)^6 + \left(r_j^{0}\right)^6}{2} \right)^{1/6}$$

$$\epsilon_{ij} = 2\sqrt{\epsilon_i \cdot \epsilon_j} \left(\frac{(r_i^0)^3 \cdot (r_j^0)^3}{(r_i^0)^6 \cdot (r_j^0)^6} \right)$$

The electrostatic interaction is represented using atomic partial charges. To make the charge parameters transferable, bond-increments δ_{ij}, which represent the charge separation between two valence-bonded atoms i and j, are used in the force field as parameters. For atom i, the partial charge is the sum of all charge bond increments δ_{ij}:

$$q_i = \sum_j \delta_{ij}$$

where j represents all atoms that are valence-bonded to atom i. This is sufficient to reflect the charge transfer effect.

The large number of valence parameters required for force fields mainly come from quantum mechanical (QM) data, which are now relatively easy to obtain; a high data/parameter ratio ensures a good determination of valence parameters. The non-bonding parameters of the electrostatic term and van der Waals (VDW) term are mainly calculated by QM. With the more robust simulation techniques of today, the liquid properties calculated agree well with those obtained in the original COMPASS developments. Except for a few cases, which might be due to insufficient sampling or inaccuracy in calculation of the electrostatic energies, limited by the simulation capacity 20 years ago, the same liquid densities and enthalpies of vaporization were obtained for most molecular liquids. This verifies that the original quality of COMPASS force field is preserved with the new extensions. The Simulation Conditions section in Ref. [2], as well as other references[4-7], indicate that the COMPASS II force field is suitable for this system.

[1] Huai, Sun, Zhao, et al. COMPASS II: extended coverage for polymer and drug-like molecule databases[J]. Journal of Molecular Modeling, 2016.

[2] Sun H. COMPASS: an ab initio force-field optimized for condensed-phase applications overview with details on alkane and benzene compounds. J Phys Chem B 1998,102(38):7338–7364.

[3] Yang J , Ren Y , Tian A M . COMPASS force field for 14 inorganic molecules, He, Ne, Ar, Kr, Xe, H-2, O-2, N-2, NO, CO, CO_2, NO_2, CS_2, and SO_2, in liquid phases[J]. Journal of Physical Chemistry B, 2000, 104(20).

[4] Bouazizi S, Nasr S, et al. Local order in aqueous NaCl solutions and pure water: X-ray scattering and molecular dynamics simulations study.[J]. Journal of Physical Chemistry B, 2006, 110(46):23515.

[5] Bouazizi S , Nasr S . Structural investigations of high concentrated aqueous LiCl solutions: X-ray scattering and MD simulations approach[J]. Journal of Molecular Structure, 2008, 875(1-3):121-129.

[6] H. Zhu, X. Zhuang, X. Meng, M. Wang, X. Wang, F. Li, J. Yuan. Concentration-dependent structure of mixed $(NH_4)_2SO_4$ and K_2SO_4 aqueous solutions using the X-ray diffraction, Raman spectroscopy and molecular dynamics simulations, Vib Spectrosc. 116 (2021). https://doi.org/10.1016/j.vibspec.2021.103292.

X. Meng, X. Zhuang, Y. Fu, J. Yuan, S. Li, S. Chen, Local structure of NH_4Cl solution and its correlation with osmotic coefficient by molecular dynamics simulation and Raman spectroscopy, Vib Spectrosc. 111 (2020). https://doi.org/10.1016/j.vibspec.2020.103155.
